# Outcomes and factors associated with survival of patients with HIV/AIDS initiating antiretroviral treatment in Liangshan Prefecture, southwest of China: A retrospective cohort study from 2005 to 2013

**DOI:** 10.1097/MD.0000000000003969

**Published:** 2016-07-08

**Authors:** Guang Zhang, Yuhan Gong, Qixing Wang, Ling Deng, Shize Zhang, Qiang Liao, Gang Yu, Ke Wang, Ju Wang, Shaodong Ye, Zhongfu Liu

**Affiliations:** a National Center for AIDS/STD Control and Prevention, China CDC, Beijing; b Liangshan Prefecture Center for Disease Control and Prevention, Xichang; c Fengtai District Center for Disease Control and Prevention, Beijing, China.

**Keywords:** AIDS, antiretroviral treatment, survival analysis

## Abstract

Human immunodeficiency virus (HIV)–positive cases have been reported among people who injected drugs in Liangshan Prefecture in southwest of China since 1995 and Liangshan has become one of the most seriously affected epidemic areas in China. In 2004, several patients with HIV/acquired immunodeficiency syndrome (AIDS) initiated antiretroviral treatment (ART) at the Central Hospital of Liangshan Prefecture. From 2005 to 2013, the number of patients receiving ART dramatically increased.

We conducted a retrospective cohort study to analyze the long-term survival time and associated factors among patients with HIV/AIDS who received ART in Liangshan Prefecture for the first time. Data were collected from the Chinese AIDS Antiretroviral Therapy DATAFax Information System. A life table and the Kaplan–Meier and Cox proportion hazard regression were used to calculate the survival time and its associated factors, respectively.

Among 8310 ART-naïve patients with HIV/AIDS who initiated ART, 436 patients died of AIDS-related diseases, and their median time of receiving ART was 15.0 ± 12.3 months, whereas 28.7% of them died within the first 6 months after treatment. The cumulative survival rates of those receiving ART in 1, 2, 3, 4, and 5 years were 97.1%, 93.4%, 90.6%, 88.8%, and 86.0%, respectively. Multivariate Cox regression analysis showed that male patients on ART were at a higher risk of death from AIDS-related diseases (adjusted hazard ratio [AHR] = 1.5, 95% confidence interval [CI]: 1.1–2.1) than female patients. Patients infected with HIV through injection drug use (IDU) were at a higher risk of death (AHR = 1.6, 95% CI: 1.2–2.2) than those infected through heterosexual transmission. Patients with a baseline CD4 cell count <50/mm^3^ (AHR = 9.8, 95% CI: 6.0–15.9), 50–199/mm^3^ (AHR = 3.3, 95% CI: 2.3–4.6), and 200–349/mm^3^ (AHR = 1.7, 95% CI: 1.2–2.3) were at a higher risk of death than those with a CD4 cell count ≥350/mm^3^.

ART prolonged survival time of patients with HIV/AIDS and improved their survival probability. Patients with HIV/AIDS should be consistently followed up and the CD4 T-cell count regularly monitored, and timely and early antiretroviral therapy initiated in order to achieve a better survival rate.

## 1 Introduction

China health authorities estimated that the number of people living with human immunodeficiency virus (PLHIV)/acquired immunodeficiency syndrome (AIDS) would account for 0.033% of China's total population by the end of 2013.^[1]^ Human immunodeficiency virus (HIV)/AIDS prevalence in China remains generally low, but the epidemic is severe in some areas, such as provinces located in Southwest China, including Yunnan, Sichuan, and Guizhou. Gradual progression of HIV to AIDS has resulted in an increase of the AIDS-related deaths. In December 2003, the government of China announced the “Four Frees and One Care” policy^[2]^ to ensure the accessibility of antiretroviral treatment (ART) to poor urban and all rural PLHIV. Since 2003, China has scaled up free ART for patients with HIV/AIDS, resulting in an increased ART coverage and decreased HIV-related mortality. By the end of 2011, a total of 150,692 patients with HIV/AIDS were treated through China's National Free Antiretroviral Treatment Program, and 122,613 of them are still on treatment.^[3]^ The national “12th Five-Year” Action Plan for Containment and Prevention of HIV/AIDS set goals for reducing new HIV infections by 25% and reducing mortality from AIDS-related causes by 30% (2010 baseline). In order to reach these goals, the State Council of China issued the “State Council on Further Strengthening the Work on Response to HIV/AIDS” in February 2011, requiring expanded antiretroviral therapy (ART) coverage to improve treatment accessibility.

Free ART has been provided in China since 2003. However, the scale-up of ART service in China encountered many challenges in terms of coverage and quality, particularly at the county level in western China^[4]^ due to patient sociodemographic diversity, lack of medical resources, and treatment delivery gaps.

As one of the most serious AIDS epidemic areas in China, HIV positives were reported among the injection drug users in Liangshan Prefecture of China in 1995. HIV spread in Liangshan through needle sharing among people who injected drugs during the earlier period of the epidemic. Since then, Liangshan has become one of the heavily HIV-affected epidemic regions in China, with increasing HIV-related morbidity and mortality. ART reduces morbidity from HIV infection, decreases death from HIV/AIDS-related diseases,^[5,6]^ improves patients’ quality of life, and restores patients’ participation in social and family functions.^[7]^ Thus, Liangshan Prefecture began to provide free antiretroviral treatment for PLHIV in accordance with standard treatment procedures in 2005. As only limited studies have been published on HIV/AIDS-related mortality and its predictors among the patients on ART in Liangshan, we conducted this retrospective cohort study to shed light on survival time and its associated factors among patients with HIV/AIDS who were ART naïve prior to ART initiation from 2005 to 2013.

## 2 Materials and methods

### 2.1 Study area

Liangshan Yi autonomous prefecture was and still is a remote, impoverished, and mountainous area, located in Southwest China. Liangshan had a population of 4.97 million people at the end of 2013, of which 50.7% (2.52 million) were of Yi ethnic minority making it the largest Yi population in China. Liangshan, 60,400 km^2^ in area, has jurisdiction over 17 counties and cities, which are Xichang, Zhaojue, Butuo, Puge, Jinyang, Meigu, Yianyuan, Ganluo, Huili, Huidong, Dechang, Xide, Leibo, Mianning, Yuexi, Muli, and Ningnan. Heath care including facilities, equipment, and personnel had been underdeveloped. The China government and international society has exerted great efforts on ART and health intervention services to Liangshan since 2004. ART was started among a very few patients with HIV/AIDS in 1 central hospital of Liangshan in 2004, and most of those patients were scattered in the remote rural areas and could not reach the treatment services on a timely basis. The lack of awareness and knowledge of HIV infection, restricted resources, and high treatment costs were also barriers to reach ART services. The first group of 4 ART-designated hospitals was set up in 2008 at county level. By 2013, a total of 18 designated hospitals were available to provide ART services at the county level in Liangshan. All ART-designated hospitals have provided free ART and physical check for patients with HIV/AIDS since September 1, 2010.

### 2.2 Study design and target population

A retrospective cohort study design was used to enroll patients with HIV/AIDS receiving ART in the 18 designated hospitals for patients with AIDS in Liangshan between January 2005 and December 2013. Among the 8602 cases enrolled from 2005 to 2013, a total of 8310 cases were included in the study. All 8310 patients were at least 18 years of age, ART naïve prior to ART, and based on the treatment criteria of the “National AIDS Free Antiviral Treatment Manual.”^[8]^ Physical and clinical examinations were provided on ART enrollment. Hospital staff recorded information from each follow-up visit (every 6 months), including date of death, cause of death, and dropout or transfer to another medical facility.

### 2.3 Data collection

Demographic data, time of HIV diagnosis, date of ART initiation, death, and medical characteristics were downloaded from the Chinese AIDS Antiretroviral Therapy DATAFax Information System, which is attached to the National Information Management System on HIV/AIDS Prevention and Control, and eligibility of patients was checked. We grouped the patients into 5 categories including constitutional (fever or lymphadenopathy), pulmonary (cough, dyspnea, chest pain, or night sweats), gastrointestinal (nausea, vomiting, or diarrhea), skin or mucosal (rash, thrush, or oral hairy leukoplakia), and neurological (headache or visual changes). If there were difficulties in classification in diagnosis of opportunistic infections in some designated hospitals, signs and symptoms collected at the first physical examination served as surrogates.

The ART initiation date was counted as the first follow-up visit of the research, and the follow-up deadline was December 31, 2013. If a patient died from HIV/AIDS-related causes, the date of death was recorded as the date of the outcome. If a patient died from an illegal drug overdose, suicide, traffic accident, other diseases, or other causes of death, or was lost to follow-up, the case was censored at the date of the last record. If the patient survived through the deadline, December 31, 2013, then that was his/her date of censorship. The assumed date of lost to follow-up was defined as the last follow-up visit date adding 3 months, which is half of the interval of follow-up, that is, 6 months. Date of death was verified based on patients’ hospital record, or national residence registration system if death occurred outside hospitals, or was reported by the decedent's relatives.

### 2.4 Ethical consideration

Ethnical approval (no.: X140617339) for this study was obtained from the Institutional Review Board of the National Center for AIDS/STD Control and Prevention, Chinese Center for Disease Control and Prevention. Permission to access ART registers and database was given by the Liangshan Prefecture Health Bureau and each of the 18 designated hospitals that provided ART services for patients with HIV/AIDS in Liangshan. No personally identifiable information was seen or used during data analysis.

### 2.5 Data analyses

The database was set up via EpiData 3.0 for windows (EpiData Association, Odense, Denmark) and transferred and analyzed via SPSS version 17.0 (SPSS Inc, Chicago, IL). Categorical data were summarized by frequencies. Continuous variables were summarized using medians and respective standard deviations (SDs). Median survival time of patients receiving ART services was calculated by Kaplan–Meier estimator/methods as a point where 50% of the patients were still alive. Survival time after ART initiation was calculated by different categories of predictors, and differences in survival time between these predictors were examined by using the log-rank test of Kaplan–Meier. Univariate factors with a *P* value <0.1 were included into Cox proportional hazard models. Cox proportional hazard models were used to estimate the hazard ratio of death between levels of confounders. Unadjusted and adjusted hazard ratios (AHRs) with their 95% confidence interval (CI) were reported. All the tests were 2-tailed, and the type 1 error rate was set at 5%.

## 3 Results

### 3.1 Baseline characteristics

Among 8310 patients receiving ART services, the median age at ART initiation was 34.6 ± 9.1 years, with an age range from 18 to 89 years of age. Age at initiation did not significantly differ between males and females (males 34.6, SD ± 9.1 years, vs. females 34.5, SD ± 9.1 years). The patient's demographic and medical characteristics by enrollment year to initial ART services are shown in Table 1. Among 8310 patients, the proportion of male patients accounted for 73.1% (6073), and female patients 26.9% (2237), 82.5% (6856/8310) were married, 10.5% (872/8310) were single, and 7.0% (582/8310) were divorced or separated.

**Table 1:**
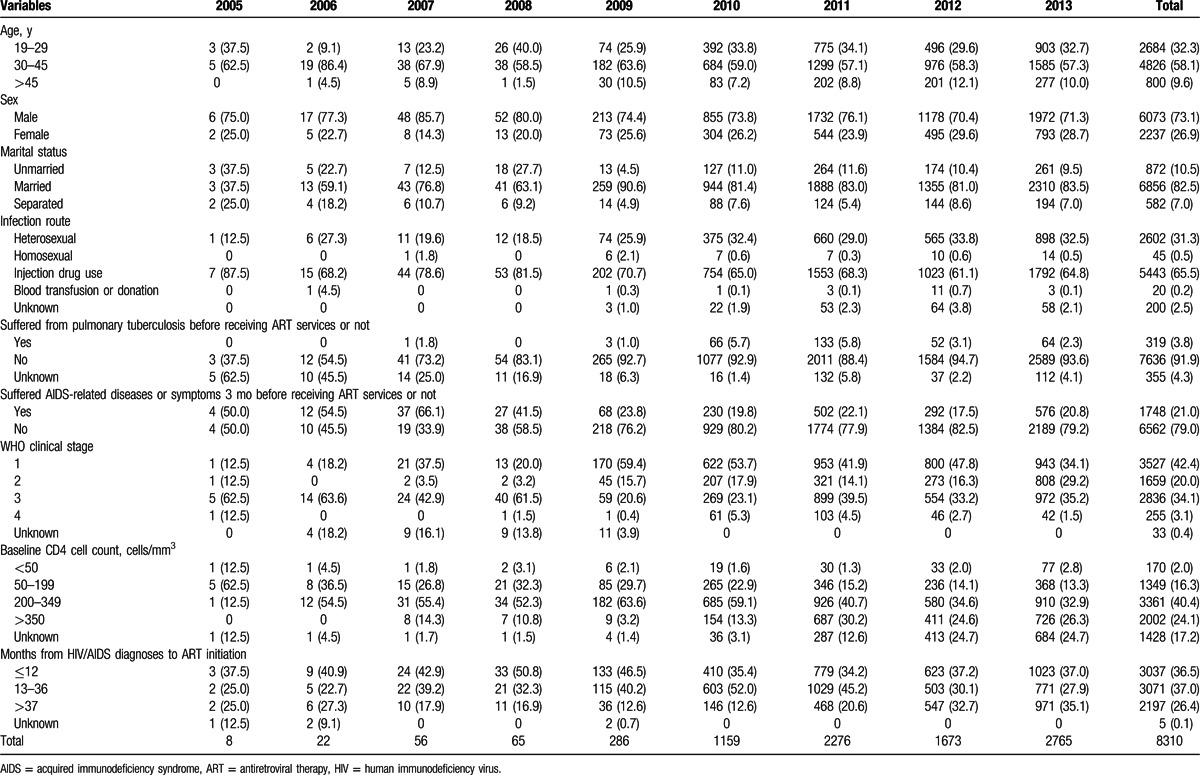
Distribution of sociodemographic and medical characteristics by ART initiation year among patients receiving ART services in Liangshan Prefecture.

About two-thirds of patients, 65.5% (5443/8310), were infected through IDU, 31.3% (2602/8310) through heterosexual transmission, 0.5% (54/8310) through homosexual transmission, 0.2% (20/8310) through blood transfusion or paid plasma donation, and 2.4% (200/8310) of the transmissions were unknown. The median time from diagnosis of HIV infection to ART initiation was 24.7 ± 21.7 months. The longest was 157 months. A total of 36.6% (3037/8310) of patients initiated ART at <12 months after HIV/AIDS diagnosis, 37.0% (3071/8310) enrolled between 13 and 36 months after diagnosis, and 26.4% (2197/8310) initiated at >37 months after diagnosis.

A total of 3.8% (319/8310) of patients suffered from pulmonary tuberculosis (TB) before receiving ART services, 89.3% (285/319) of whom received anti-TB therapy. A total of 21.0% (1748/8310) of patients suffered from AIDS-related diseases or symptoms 3 months before starting ART. In terms of WHO clinical staging, 42.4% (3527/8310) of patients started treatment in stage I, 20.0% (1659/8310) in stage II, 34.1% (2836/8310) in stage III, and 3.1% (255/8310) in stage IV. The overall median CD4 cell count for 6884 patients who received CD4 testing when enrolling in ART services was 321.2 ± 200.3 cells/mm^3^. Among those (6884) having a CD4 cell count, 2.5% (170) of the patients were <50 cells/mm^3^, 19.6% (1349) 50 to 199 cells/mm^3^, 48.8% (3361) 200 to 349 cells/mm^3^, and 29.1% (2004) >350 cells/mm^3^ at the start of ART.

The number of patients initiating ART significantly increased by year from 8 cases in 2005 to 2765 cases in 2013. The proportions of patients between 30 and 45 years of age decreased from 72.1% (62/86) in the first period, 2005 to 2007, to 57.5% (3860/6714) in the third period, 2011 to 2013 (*P* < 0.001). The proportion of patients suffering from AIDS-related diseases or symptoms 3 months before receiving ART services decreased from 61.6% (53/86) in the first period to 20.4% (1370/6714) in the third period (*P* < 0.001), and patients with a baseline CD4 cell count <350 cells/mm^3^ decreased from 87.2% (75/86) in the first period to 52.2% (3506/6714) in the third period (*P* < 0.001). A majority of patients (51.2%) who initiated ART in the first period were in advanced WHO clinical stages 3 and 4, but only 38.9% (2620/6714) of patients in the third period were in advanced WHO clinical stages 3 and 4, and the difference was significant (see Table 1).

### 3.2 Survival analysis

By the end of 2013, a total of 76.0% (6320/8310) of patients were still receiving ART services, 13.3% (1101/8310) had dropped out of ART services, 2.5% (207/8310) were lost to follow-up, 5.3% (436/8310) had died of AIDS-related diseases, and 3.0% (246/8310) had died of other causes. The overall survival rate of the patients was found to be 96.6/100 person-years. Among the patients who died of AIDS-related diseases during the follow-up period, their average treatment time was 15.0 ± 12.3 months. A total of 28.7% (125/436) died in their first 6 months of ART initiation, 16.3% (71/436) died between 6 and 12 months of ART initiation, and 55.1% (240/436) died after 12 months of starting ART.

Among the patients who died in the first 6 months from ART initiation, 51.2% (64/125) were in WHO clinical stages III and IV, and baseline CD4 cell counts for 59.6% (65/109) were <200 cells/mm^3^. Among the patients who died between 6 and 12 months after ART initiation, 42.3% (30/71) were WHO clinical stage III or IV, and 45.5% (30/66) of CD4 cell counts were <200 cells/mm^3^. Among the patients who died after 12 months from starting ART, 32.1% (77/240) were in WHO clinical stage III or IV, and 36.5% (81/222) of CD4 cell counts were <200 cells/mm^3^.

**Figure 1:**
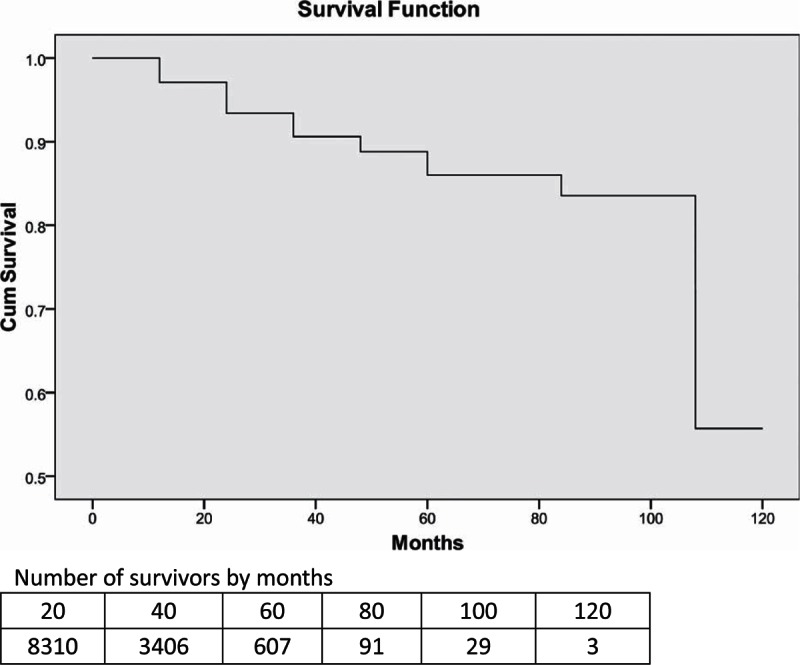
Survival curve for patients with HIV/AIDS receiving ART services in Liangshan Prefecture. AIDS = acquired immunodeficiency syndrome, ART = antiretroviral therapy, HIV = human immunodeficiency virus.

**Figure 2:**
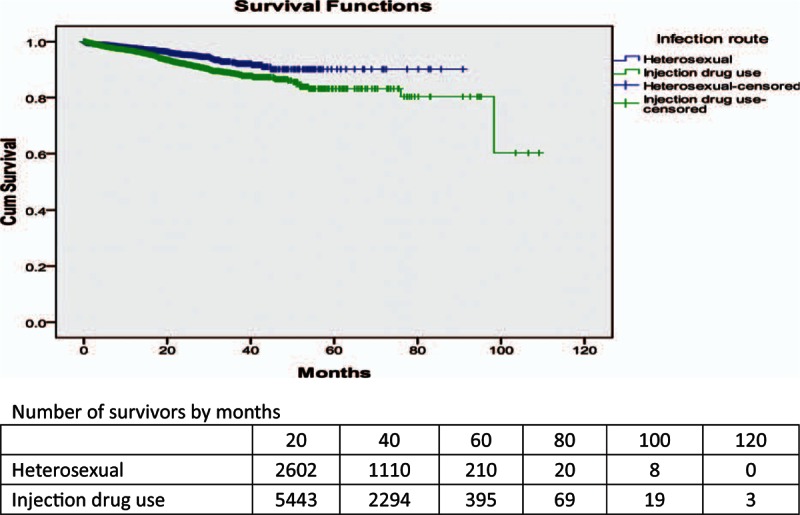
Survival curves for patients with HIV/AIDS receiving ART services by different infection routes in Liangshan Prefecture. AIDS = acquired immunodeficiency syndrome, ART = antiretroviral therapy, HIV = human immunodeficiency virus.

Cumulative proportions of survival of the study population are shown by 12-month intervals in Table 2. By December 31, 2013, the overall median survival time for patients receiving ART services was 94.3 (95% CI: 90.0–98.6) months. The cumulative proportion surviving over the 1st year was 97.1%, 93.4% over the 2nd year, 90.6% over the 3rd year, 88.8% over the 4th year, 86.0% over the 5th year, and 55.7% over the 10th year (see Table 2; Fig. 1). Comparing the survival curves for patients with HIV/AIDS receiving ART services by 2 different transmission modes, we found that the cumulative proportion of surviving patients infected via heterosexual route was higher than that of those infected via IDU (see Fig. 2).

**Table 2:**
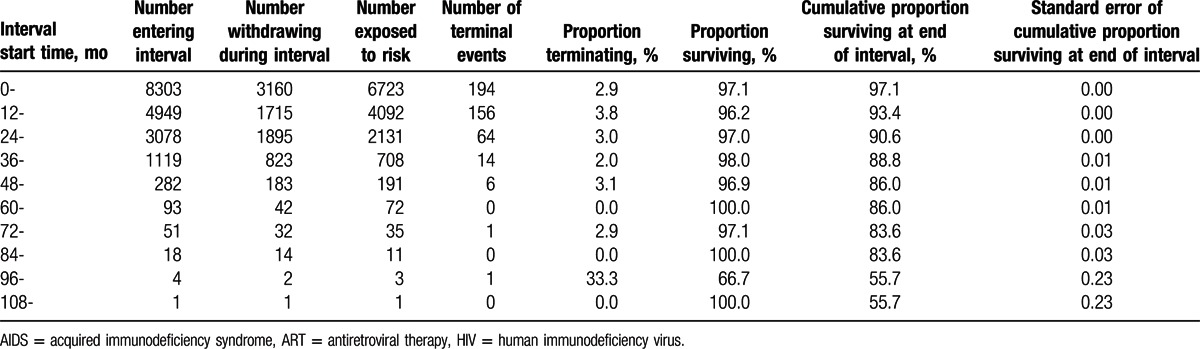
Survival analysis of patients with HIV/AIDS receiving ART services in Liangshan Prefecture.

By comparing patients receiving ART services by different sociodemographic and medical characteristics in Table 3, we found that female patients with HIV/AIDS had a longer survival time (100.4 months, 95% CI: 98.7–102.2) than male patients (91.5 months, 95% CI: 86.3–96.7), patients with HIV/AIDS who stared treatment in WHO clinical stage I, II, or III had a longer survival time than those in advanced WHO clinical stage (IV) (44.1 months, 95% CI: 41.7–46.5), and patients infected through IDU had a longer survival time (92.3 months, 95% CI: 87.8–96.8) than other infection routes. Also, patients with a baseline CD4 cell count >50 cells/mm^3^ survived longer (82.6 months, 95% CI: 79.6–86.4) than patients whose baseline CD4 cell count was <50 cells/mm^3^ (37.6 months, 95% CI: 32.7–42.4), and patients who had not suffered from pulmonary TB had longer survival times (70.3 months, 95% CI: 66.2–74.3) than those who had suffered from pulmonary TB (92.0 months, 95% CI: 85.5–98.6) (see Table 3).

**Table 3:**
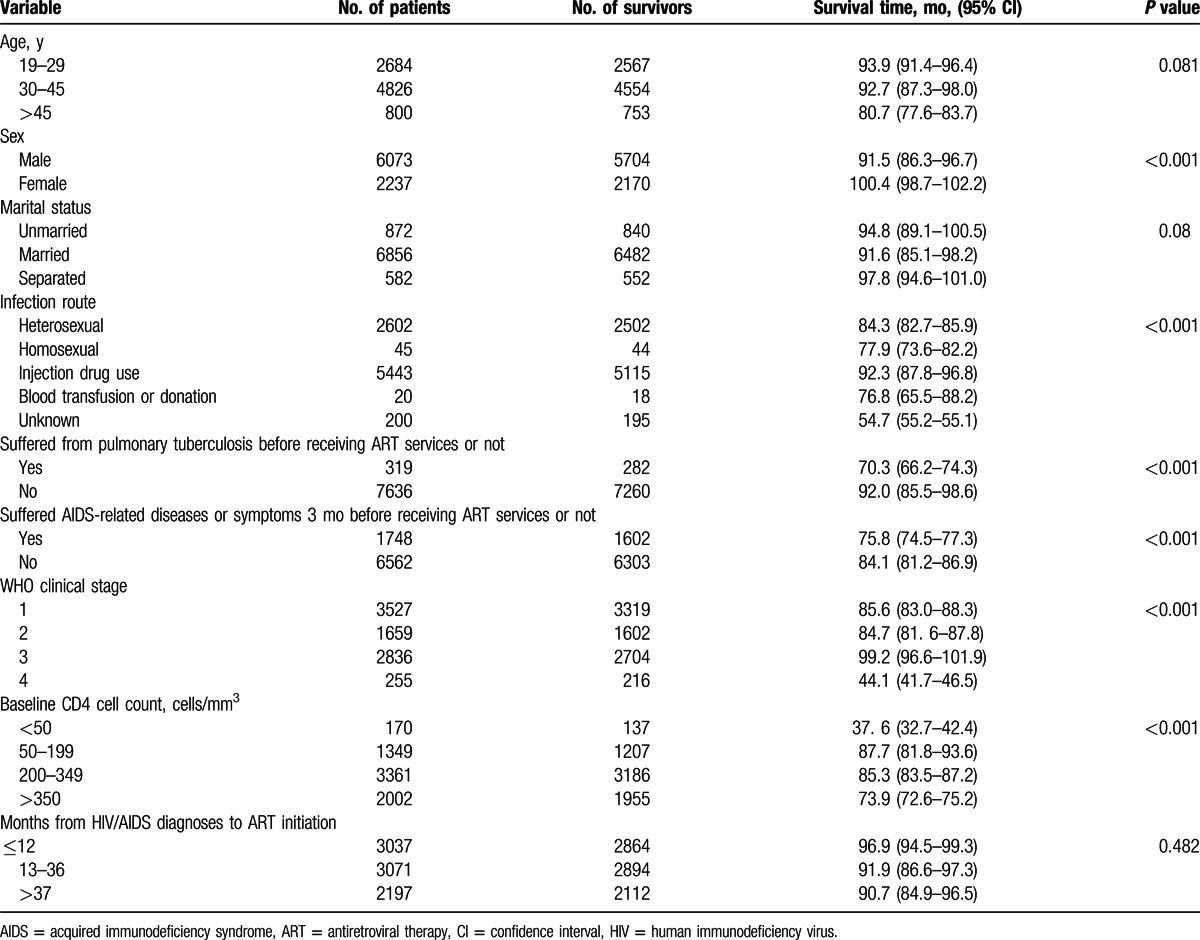
Survival time by sociodemographic and medical characteristics of patients with HIV/AIDS receiving ART services in Liangshan Prefecture.

### 3.3 Predictors of survival time

Male patients on ART had 1.5 times the rate of death (AHR = 1.5, 95% CI: 1.1–2.1) than female patients in Liangshan Prefecture (Table 4). Married patients had 1.7 (AHR = 1.7, 95% CI: 1.1–2.5) times the death hazard rate compared to those unmarried when starting ART. Patients infected with HIV through IDU were at a higher risk of death (AHR = 1.6, 95% CI: 1.2–2.2) compared to those infected through heterosexual transmission.

**Table 4:**
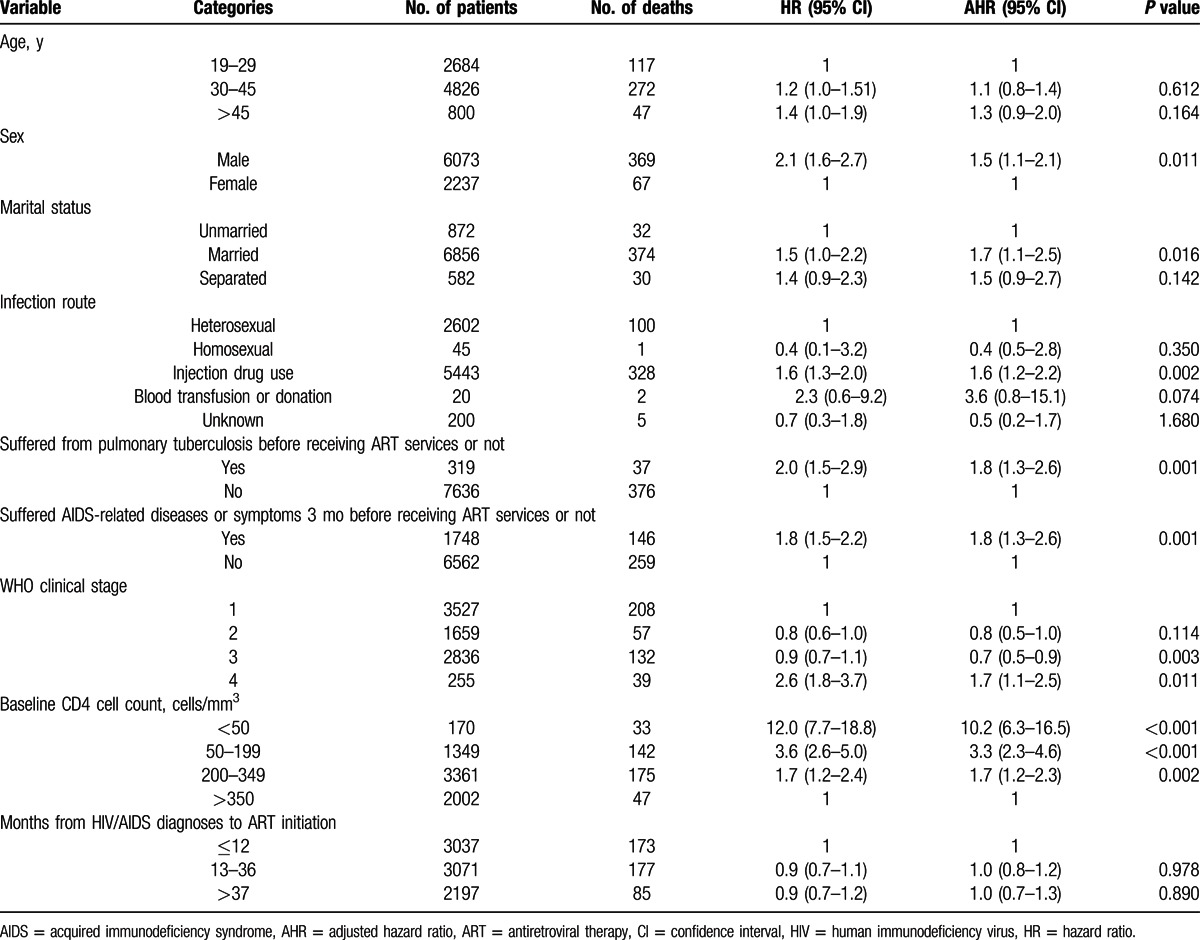
Unadjusted and AHRs of factors of survival time of patients with HIV/AIDS receiving ART services in Liangshan Prefecture.

Pulmonary TB and AIDS-related diseases or symptoms 3 months before receiving ART services were found to be significant predictors of mortality. Patients with pulmonary TB and AIDS-related diseases or symptoms had a higher death hazard rate compared to those who did not suffer from these diseases (AHR = 1.8, 95% CI: 1.3–2.6, and AHR = 1.8, 95% CI: 1.3–2.6, respectively).

Compared to those starting treatment with >350 cells/mm^3^, the death hazard rate was about 10 times higher (AHR = 9.8, 95% CI: 6.0–15.9) among patients starting ART with a CD4 cell count <50 cells/mm^3^, 3 times higher (AHR = 3.3, 95% CI: 2.3–4.6) among those with a CD4 cell count between 50 and 199 cells/mm^3^, and 2 times higher (AHR = 1.7, 95% CI: 1.2–2.3) among those with a CD4 cell count between 200 and 349 cells/mm^3^.

WHO classification clinical stage at ART enrollment was a predictor of mortality among the patients receiving treatment. The patients at WHO stage 4 when starting ART had twice the death rate of patients at WHO stage 1.

## 4 Discussion

This study analyzed survival outcomes and explored the associated factors of survival time among patients with HIV/AIDS receiving ART in Liangshan for the first time. The median survival time after ART initiation among the patients during the study period was 94 months. This result was higher than those of the studies in Tanzania, Botswana, Uganda, and South Africa.^[9–12]^ ART has been associated with prolonging survival time of patients with HIV/AIDS. However, survival time of patients with HIV/AIDS receiving ART services varied in different countries and regions, which depended on adherence of therapy, baseline CD4 cell count, demographic characteristics of the patients, and coinfection of diseases.^[13]^ We found that the first year's cumulative proportion of surviving patients with HIV/AIDS in Liangshan was similar to the cumulative mortality rate from a triple-drug ART initiation study in British Columbia, Canada, in 2000,^[14]^ but lower than that of studies of high-income countries.^[6]^ Results of other years were similar to the survival analysis study of 3013 cases from Dehong Prefecture of Yunnan Province, China,^[15]^ in which 2-, 3-, 4-, and 5-year survival rates were 94%, 93%, 92%, and 92%, respectively, but higher than those of patients treated in Botswana's national ART program.^[11]^

Compared with other years, mortality and morbidity of the first year after ART initiation were higher, especially within the first 6 months.^[16]^ Among patients who died from AIDS-related diseases, the majority of deaths occurred within the first 12 months after initiating ART, which was similar to the results of a study in southern India,^[17]^ and lower than the study in Botswana in which approximately 86% of all deaths occurred within the first year.^[11]^ Most of the patients who died in the first year after starting ART were in advanced clinical stages of the disease or WHO clinical stage III or IV, and their CD4 cell count was <200 cells/mm^3^. The first 12 months of therapy is critical in reducing the chance of dying from AIDS-related diseases. Thus, earlier CD4 cell count testing, follow-up of patients, and clinical observation should be strengthened and especially focused on patients with a lower CD4 count and advanced WHO clinical stages.^[18,19]^

The overall basic median CD4 cell count (321.2 cells/mm^3^) was higher than the figures of the MASA Program implemented in Botswana from 2002 to 2010,^[20]^ and that of a 5-year retrospective cohort study during 2004 to 2009 in Tanzania,^[9]^ but similar to the baseline CD4 cell count of newly identified HIV cases in China in 2012.^[21]^ The proportion of the patients starting ART with a CD4 cell count <200 cells/mm^3^ (22.1%) and the proportion of patients with advanced WHO clinical stage 3 or 4 (37.3%) were significantly lower than sub-Saharan Africa countries, such as Tanzania and South Africa.^[9,18]^ With the criteria of initiating ART on a CD4 count lifting from <200 to <350 cells/mm^3^ and the policy of scaling up free ART services promotion in rural areas of Liangshan during the period of the study,^[22]^ more patients were found, enrolled to ART services, and followed up. The basic median CD4 cell count of the patients starting ART increased, and the proportion of the CD4 count <200 cells/mm^3^ and the patients with advanced WHO clinical stage at ART initiation decreased over the years.

The results of the survival analysis showed that the risk of the male patients with HIV/AIDS who received ART dying from AIDS-related diseases was higher than that of the female patients, similar to the study of Tanzania^[9]^ and the aggregation figure of meta-analysis of observational studies in low- and middle-income counties.^[19]^ The reason for this is likely to be that female patients need to take care of their family and children as usual, stay in their hometown, and then better adhere to the treatment delivered by community-based facility compared to male patients, who are usually employed as migrant workers, fail to receive timely health care and interventions, and are more likely to drop out of the ART services system. These results were similar to the results of the study conducted in China between 2006 and 2012.^[21]^ A study on a home-based AIDS care program that provided ART and other AIDS care, prevention, and support services for patients with HIV/AIDS in a resource-limited rural African setting also showed good adherence and response to ART.^[23]^

Patients receiving ART who were infected with HIV through IDUs in Liangshan had higher morbidity than those patients infected by heterosexual transmission, which was similar to the results of Dehong Prefecture in Yunnan Province in China.^[15]^ This may be because of poor adherence of treatment among intravenous drug users and physical conditions with coinfection diseases such as hepatitis C at the start of ART for long-term injection drug user.^[24]^ An earlier study in the United States indicated that the risk of death was also greater among injection drug users than among men who have sex with men.^[25]^ Some studies from Europe and North America^[26,27]^ also showed that infection through IDU was an independent predictor for poorer outcome of patients with HIV/AIDS in ART Cohort Collaboration.

Some HIV cohort studies demonstrated that starting ART at a higher CD4 cell count would reduce morbidity and avert the high mortality rates among patients with advanced HIV disease.^[28]^ We found that patients whose CD4 cell count was <350 cells/mm^3^ had a higher risk of death compared to patients with a CD4 cell count ≥350 cells/mm^3^. The study showed that the CD4 cell count was an independent predictor of AIDS progression, and was also in line with the other research results,^[11,12,14]^ which indicated that AIDS progression to death was clustered among patients starting therapy with a CD4 cell count <350 cells/mm^3^.^[29]^ It underscores early ART initiation and regular CD4 cell count testing to monitor the prognosis of patients with HIV/AIDS receiving ART. Because of social or economic reasons, PLHIV may not receive adequate care or be aware of the status of infection and institute treatment, and the diagnosis of AIDS may be delayed,^[25,30]^ and cause a later initiation of ART at a low CD4 cell count (even <200 cells/mm^3^).^[10]^ We also found that patients who started ART with WHO clinical stage 4 suffered from pulmonary TB or AIDS-related diseases, and patients who had symptoms 3 months before receiving ART services had a higher death hazard rate compared to those with WHO clinical stage 1 and were disease or symptom free. Patients stating ART with an advanced disease stage as defined by WHO stage 4 had an increasing risk of immune reconstruction syndrome and death.^[9]^ TB and wasting syndrome appeared to be the most common causes of early mortality.^[19]^ We should prevent and manage opportunistic infections such as TB, and offer better pre-ART care to reduce early death of patients with HIV/AIDS receiving ART, especially among patients with CD4 cell counts <200 cells/mm^3^. However, a prospective, randomized, placebo-controlled trial in Africa on early initiation of ART for HIV-positive patients with TB concluded that ART can be delayed until after completion of 6 months of TB treatment for patients with TB who have a CD4 cell count of >220 cells/mm^3^.^[31]^

There are a few limitations in this study. First, because the ART information system (National Information Management System on HIV/AIDS Prevention and Control) was not established in Liangshan until 2005, the previous information regarding ART services was not involved in the cohort study. Although the number of these patients was small, their outcomes of ART might slightly impact the overall survival time. Second, the exact dates for the patients lost to follow-up were not collected, and we assumed the median time between the last 2 follow-up dates as the proxy of the date of lost to follow-up. The survival time for patients incurred a recall bias when survival analysis was carried out. Third, AIDS-related diseases were recorded by the lay medical staff based in villages and towns where the patients first enrolled, and their knowledge of diagnosis was limited. Most common signs and symptoms were categorized simply as follows: fever, pulmonary (cough, dyspnea, chest pain, night sweats, or lymphadenopathy), gastrointestinal (nausea, vomiting, or diarrhea), skin or mucosal (rash, thrush, or oral hairy leukoplakia), and central nervous system (headache or visual changes).^[5]^ This might underestimate the impacts of AIDS-related diseases on the mortality of the patients when starting ART. Finally, overall time from HIV diagnosis to receiving ART partly depended on the result and time of CD4 cell count testing.^[32]^ So some patients with lower CD4 cell counts later initiated ART, and survival time of these patients was underestimated.

Therefore, in order to reduce deaths caused by AIDS, PLHIV in Liangshan, especially those who were infected through IDU, should be timely followed up, be provided health education on adherence of treatment, and be given physical examinations, and the CD4 cell count should be monitored. ART should be timely delivered and well adhered to based on the results of follow-up, CD4 cell count testing, and physical checks.

## Acknowledgments

The authors thanks Zhijun Li, Senior Medical Officer of Global AIDS Program China Office, US Centers for Disease Control and Prevention, Beijing, China, for assisting with statistic method in the study analysis and Dr Yifei Hu, associate professor of School of Public Health, Capital Medical University, for her technical advice for manuscript revision and editorial assistance. They also thank Dr Edward C. Mignot, Shandong University, for linguistic advice.
